# Prominent Pseudo-Angiovascular invasion by benign gallbladder epithelium and bile emboli in a patient with delayed cholecystectomy due to COVID-19 positive test

**DOI:** 10.1186/s42047-022-00120-y

**Published:** 2022-09-10

**Authors:** Quinn Miller, Nishi Dave, Nikolay Popnikolov, Sidney D. Bruce, Hector Mesa

**Affiliations:** 1 Department of pathology and Laboratory Medicine, Indiana University School of Medicine, 350 W 11th St, Indianapolis, IN 46202 USA; 2 Department of General Surgery, Indiana University Health, Lafayette, IN 47905 USA

**Keywords:** Gallbladder diseases, Angioinvasion, Pseudovascular invasion, Mimics, Iatrogenic

## Abstract

Lymphovascular invasion is a hallmark of malignant neoplasms, however the presence of benign epithelium within vessels has been reported in benign processes, albeit infrequently. The proposed mechanism to explain this phenomenon entails mechanical displacement of benign epithelium into the vascular spaces during surgical manipulation or diagnostic interventions. We report a case of numerous benign epithelial vascular emboli in a cholecystectomy specimen. A 29-year-old male presented with acute calculous cholecystitis. Surgery was delayed for several weeks due to COVID-19 infection. Histologic examination of the gallbladder showed subacute cholecystitis, widespread vascular epithelial emboli with associated fibrin deposition and bile embolism supporting an in vivo process. The epithelial emboli were localized in small veins and arterioles with D2–40−/CD31+/CD34+ endothelium. The displaced epithelium showed benign cytologic features, was negative for p53 expression, and had a Ki-67 labelling index like the benign background mucosa, supporting a benign process. There was no evidence of dysplasia or malignancy in the specimen after thorough sampling. Persistent inflammation, mucosal ulceration, transmural mucosal herniation (Rokitansky-Aschoff sinuses), and protracted surgical manipulation secondary to adhesive disease are favored to be the underlying causes of this unusual histologic finding. Although we presume an uneventful outcome, clinical follow up was recommended. COVID-19 infection likely contributed to this phenomenon by causing a delay in the surgical management.

## Introduction

In surgical pathology, the microscopic presence of epithelial cells in vascular or lymphatic spaces is almost invariably associated with malignant processes and reported as lymphovascular, lymphatic, and/or angiovascular invasion. For surgeons and oncologists, reporting of this finding by pathologists is considered a risk factor for tumor relapse and metastatic disease, and often an indication for adjuvant therapy. However, the presence of benign epithelium in vessels has also been rarely reported in non-malignant processes. We describe the presence of numerous intravascular deposits of benign gallbladder epithelium in a cholecystectomy specimen of a patient who underwent a delayed cholecystectomy due to a COVID-19 positive test.

## Case presentation

A 29-year-old male presented to the emergency department with a one-week history of abdominal pain accompanied by nausea and emesis. The pain was intermittent, located in the right upper quadrant with radiation to his back, and was exacerbated by food intake. He had presented several times in the past 6 years with similar episodes. An 11 mm cholelith impacted within the gallbladder neck and multiple additional choleliths were identified on abdominal CT scan along with thickening of the gallbladder wall, consistent with acute calculous cholecystitis. The patient was started on intravenous antibiotics, morphine, and antiemetics. Surgical intervention was postponed due to a positive pre-operative COVID-19 test and the patient was discharged on oral antibiotics. Elective laparoscopic cholecystectomy was performed 4 weeks after initial presentation. During the procedure dissection was tedious due to dense adhesions and fibrous changes. Gross examination of the gallbladder revealed multiple choleliths up to 2.2 cm. The wall thickness ranged from 0.1 cm to 0.8 cm and the mucosa was roughened and focally disrupted but without discrete lesions.

### Histologic findings

**Fig. 1:**
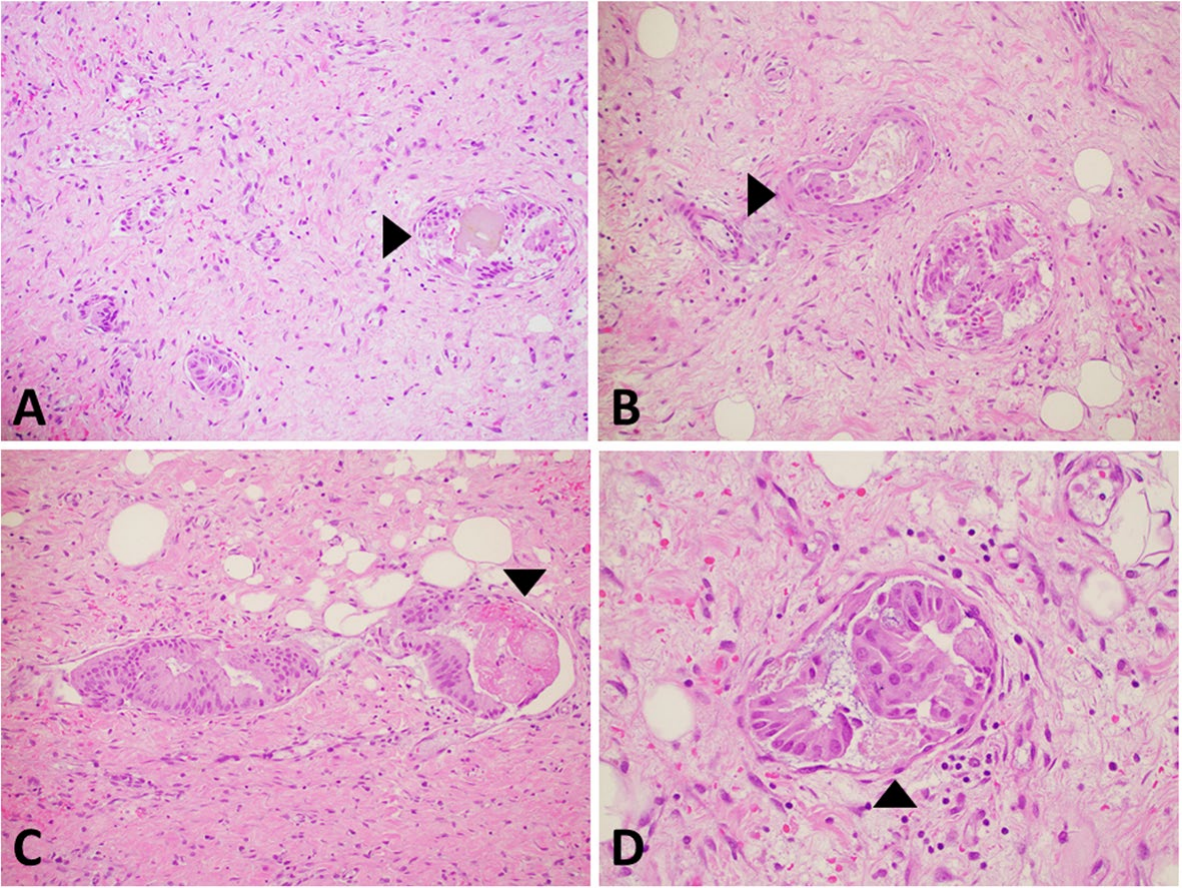
Intravascular gallbladder epithelium. **A** Low magnification image showing multiple small thin-walled vessels with epithelial fragments. The intravascular inclusion on the right (arrowhead) is associated with biliary pigment. (H&E, 10X objective). **B** Medium magnification image showing intravascular epithelium within muscular (arrowhead) and non-muscular venules. (H&E, 20X objective). **C** and **D** High magnification of intravascular epithelium conforming to the shape of the vessel and associated with a fibrin thrombi (arrowheads). (H&E, 40X objective)

**Fig. 2:**
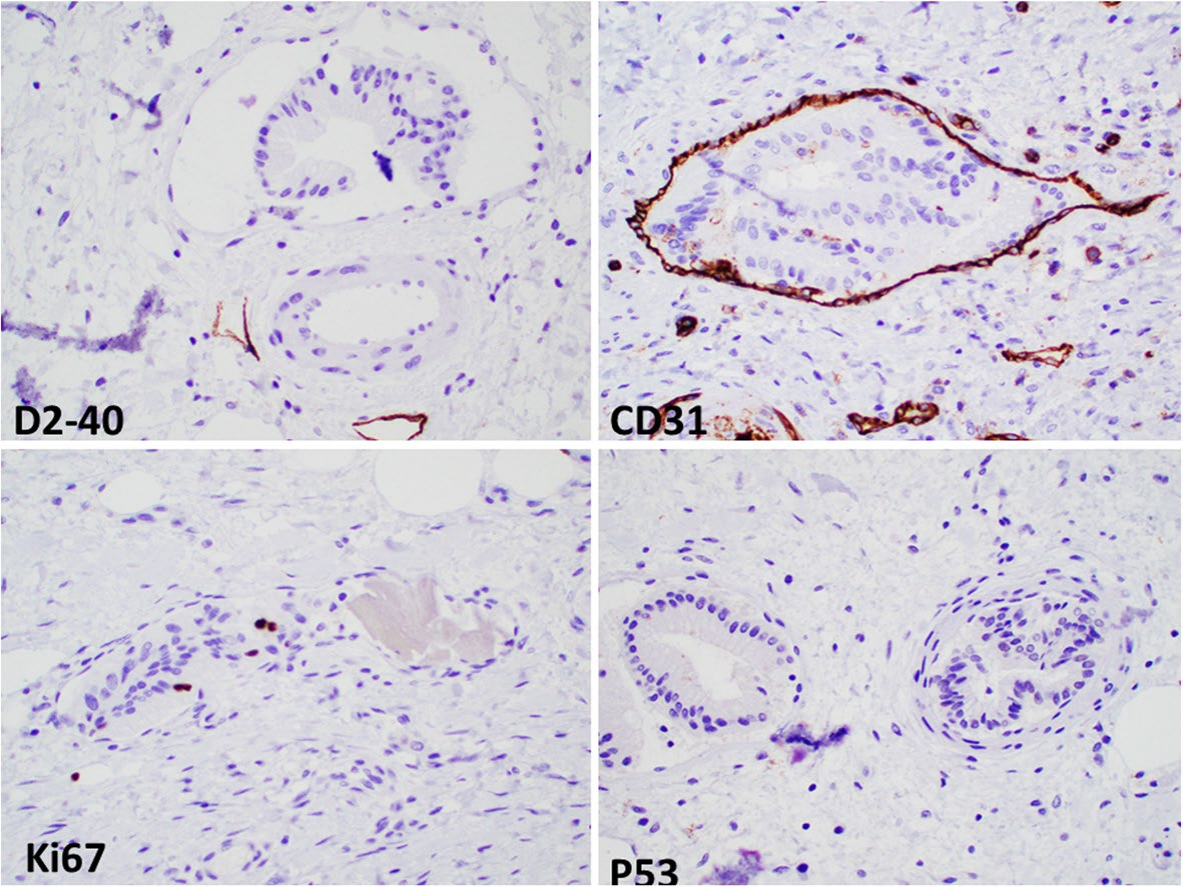
Ancillary studies. The vessels containing the epithelial groups are negative for D2–40 and positive for CD31 consistent with venules. The intravascular nests show low Ki-67 proliferative activity and are negative for P53, consistent with a benign process. (All images 20X objective)

Microscopic examination showed transmural thickening and fibrosis, prominent Rokitansky-Aschoff sinuses, subacute inflammatory infiltrate and focal ulceration. Within a localized area of the wall, numerous intramural vessels containing intraluminal fragments of biliary epithelium were discovered. Several epithelial groups were closely associated with fibrin thrombi and bile emboli supporting an in vivo process rather than a grossing artifact. The intravascular epithelium appeared benign, lacking cytologic atypia or architectural complexity. Immunostains for p53 and Ki-67 showed no difference between the intravascular epithelium and the benign background mucosa, further supporting a benign process. Generous sampling of the gallbladder performed after identifying this abnormality showed additional foci of intravascular epithelium but did not reveal evidence of dysplasia or malignancy. Some vessels within the involved regions showed organizing thrombosis and fibrin emboli. The involved submucosal vessels included thin-walled and thick-walled muscular vessels (Fig. 1). Immunostains for vascular markers showed that vessels containing the epithelium had a D2–40(−)/CD31 (+)/CD34 (variable) phenotype indicative of non-lymphatic vessels such as capillaries, venules, and arterioles (Fig. 2).

## Discussion

The presence of numerous intravascular deposits of biliary epithelium in this case was striking and unexpected, and elicited concern for an occult malignancy which prompted thorough sampling of the specimen and review of the case by several pathologists. The lack of cytologic atypia or architectural complexity in the intravascular epithelium and absence of dysplasia or precursor lesions in the specimen after thorough sampling were strong arguments against a malignant process. Additionally, the demographics of this patient: male, 29-y.o., did not conform to the epidemiology of gallbladder cancer (female, > 60-y.o.) (Pyo et al. 2020). However, a literature search of medical databases PubMed, Access Medicine, MDPI, BioMed Central using the search terms: humans, intravascular, epithelium, gallbladder, cholecystitis, did not yield any previous description of a similar phenomenon, indicating that this represents either an under-reported or very uncommon finding.

Gallbladders are common surgical specimens; pathologists are fully aware of the possibility of discovering an incidental carcinoma and the need to extensively sample specimens with any unusual histologic findings. A recent meta-analysis describes an incidence of incidental adenocarcinoma in cholecystectomy specimens of only 0.6–0.7% (Pyo et al. 2020); however, half of gallbladder cancers are diagnosed incidentally after cholecystectomy. (Shih et al. 2007) Pathologists are also aware of the existence of **“**extremely well-differentiated/minimal deviation adenocarcinomas” (EWDA) (McFarland et al. 2015; Ronnett 2016). Although these tumors usually occur in the stomach and the uterine cervix, they can rarely arise from gastric metaplastic epithelium in the biliary tract (Albores-Saavedra et al. 1999; Albores-Saavedra et al. 2012). Despite their near-benign histologic appearance, gastric EWDA are aggressive tumors with high metastatic potential (McFarland et al. 2015; Niimi et al. 2002). Histologically, these tumors are primarily recognized by their widely infiltrative growth pattern in the wall of the organs in which they arise. In our case the presence of intravascular tumor without an associated infiltrative process in the gallbladder wall was not supportive of this diagnosis. The use of the ancillary immunostains p53 and Ki-67 to recognize EWDA has been described in some studies (Ronnett 2016; Niimi et al. 2002), and prompted us to use these markers in this case. These markers are useful if an aberrant pattern of expression is identified in the tumor compared to the normal background mucosa, but do not exclude the possibility of malignancy if no abnormality is identified as in our case. Together the epidemiologic, histologic, and ancillary studies in this case were supportive of a benign process.

Several possible explanations for the observed epithelial intravascular emboli were considered: First, we considered artifactual angioinvasion: the emboli were limited to a specific region of the gallbladder wall, they were present in multiple blocks and levels and there was no epithelial displacement in non-vascular spaces to suggest that they represented displaced epithelium during grossing. The epithelium often conformed to the shape of the vascular spaces and was associated with fibrin thrombi and bile emboli supporting an in vivo process.

Second, true angioinvasion was considered: vascular infiltration not related to medical intervention, in the context of benign processes, has been described in benign proliferative processes of the breast (Eusebai and Azzopardi 1976), endometriosis (Jerman and Hey-Cunningham 2015), endosalpingiosis (Russell and Anderson 2016) and vasitis nodosa (Balogh and Travis 1985). However true angioinvasion could not explain the coexistence of epithelial and bile emboli, since bile pigment is inert and can only be passively displaced into vascular spaces.

Finally, mechanical pseudoangioinvasion due to manipulation of the gallbladder during surgery was a much logical explanation for the observed pathologic findings. Artefactual displacement of benign epithelium into lymphovascular spaces after invasive procedures has been reported in other organs such as breast (Koo et al. 2010) and uterus (Krizova et al. 2011). Benign gallbladder epithelium within lymphatics was identified in the gallbladder of a 4-year-old child with a congenital choledochal cyst and increased intraluminal biliary pressure who underwent a complex bile duct resection and reconstruction (Hirayama et al. 2009). The increased intrabiliary pressure probably played a role in this process since abnormal intrabiliary pressures leading to cholangiovenous reflux have been reported during cholangiography (Yoshimoto et al. 1989). In addition, tissue damage (gunshot wounds, medical interventions) and necrosis, leading to abnormal communications between the biliary tract and vessels, have been associated with biliary embolism (Proia et al. 1986). Several of those factors are present in our case: the procedure required an unusual time of manipulation and was described as “tedious,” requiring time-consuming takedown of dense fibrous adhesions and use of intravenous indocyanine green dye. The impacted cholelith within the cystic duct supports an existing increased intraluminal pressure that may have increased further upon manipulation and probably became at least transiently higher than the intravascular pressure, favoring the displacement of epithelium and bile into the vessels. Areas of mucosal ulceration and prominent Rokitansky-Aschoff sinuses adjacent to dilated vessels were easily identifiable, confirming proximity between epithelium and dilated medium-sized vessels. Organizing thrombi within vessels were observed, supporting abnormal intravascular pressures.

In summary, we describe a case of pseudoangiovascular invasion by benign gallbladder epithelium in a cholecystectomy specimen of a patient who had a delayed cholecystectomy due to a COVID-19 positive test. We favor that this extremely uncommon phenomenon, not previously described, is the result of mechanical, iatrogenically caused displacement of epithelium into abnormally dilated vessels due to an unusually prolonged and difficult surgical manipulation of the gallbladder, in the context of abnormal intraluminal and intravascular pressures. COVID-19 infection likely contributed to this phenomenon by causing a delay in the surgical management.

## Data Availability

Data sharing is not applicable to this article as no datasets were generated during the current study.
